# Fabrication and Characterization of Oxygenated AlN/4H-SiC Heterojunction Diodes

**DOI:** 10.3390/ma13194335

**Published:** 2020-09-29

**Authors:** Dong-Hyeon Kim, Seong-Ji Min, Jong-Min Oh, Sang-Mo Koo

**Affiliations:** Department of Electronic Materials Engineering, Kwangwoon University, 20 Kwangwoon-ro, Nowon-gu, Seoul 01897, Korea; gogomatt@kw.ac.kr (D.-H.K.); sjmin@kw.ac.kr (S.-J.M.)

**Keywords:** aluminum nitride, silicon carbide, rapid thermal annealing, Schottky barrier diodes, radio frequency sputtering, Auger electron spectroscopy

## Abstract

The effects of rapid thermal annealing (RTA) on Schottky barrier diodes (SBDs) made from oxygenated aluminum nitride (AlN) thin films deposited on a silicon carbide (SiC) substrate using radio frequency sputtering were investigated. The annealed SBD devices exhibited a 10x increase in the on/off current ratio vs. non-annealed devices for measurement temperatures ranging from 300 K to 450 K. The ideality factor, derived from the current density–voltage (J-V) characterization, increased by a factor of ~2.2 after annealing, whereas the barrier height decreased from ~0.91 to ~0.68 eV. Additionally, Auger electron spectroscopy indicated decreased concentrations of atomic oxygen in the AlN thin film, from ~36% before, to ~24% after annealing. This may have contributed to the reduced barrier height and improved on/off ratio in the annealed AlN/SiC diodes.

## 1. Introduction

Owing to its high bandgap (~6.2 eV), high breakdown voltage, high thermal conductivity and low thermal expansion, aluminum nitride (AlN) is of considerable interest for the manufacture of deep ultraviolet (DUV) lasers, LEDs and detectors [1,2]. The lattice match of AlN and SiC makes SiC a suitable substrate on which to grow AlN thin films. It is reported that the highly-oriented MoS_2_ film may act as an excellent template for guiding the growth of AlN on a 2D surface at lower temperatures and that, additionally, annealing can provide low dislocation density AlN templates [3,4,5]. AlN epilayers of high quality are commonly obtained using metal-organic chemical vapor deposition (MOCVD). AlN grown at high temperature on sapphire substrates need to be relatively thick (upwards of ~600 nm) for achieving high-quality films [6], limiting its suitability for certain device types. Sputtered thin films are known to have the disadvantage of possible degradation during deposition due to the plasma. This can be avoided by using molecular beam epitaxy (MBE), which allows highly controlled thin film growth. However, the primary disadvantage of MBE includes high operation costs and difficulty in scaling up. Hence, as the deposition of AlN thin films at low temperatures has become increasingly important, the sputtering technique is promising under circumstances where low-temperature deposition, large-scale or conformal film growth are to be readily achieved [7,8,9,10]. However, this results in a low polar field which diminishes the performance of high electron mobility transistors [1,11]. AlN thin films grown by RF-sputtering contain defects relating to oxygen impurities, resulting in impaired electrical and optical properties [12]. The theoretical modeling of oxygen in semiconductor materials is still computationally challenging. This is due to the native oxygen (e.g., binding properties, large electronegativity), which is difficult to clearly explain using conventional empirical or semi-empirical methods [13]. Annealing is an important process for manufacturing high-quality compound semiconductors such as GaAs, SiC, and AlN. Annealing reduces the number of defects in GaAs thin films by approximately two orders of magnitude. [14]. Similarly, annealing of AlN substrates and epilayers in an oxygen atmosphere results in the passivation of deep donor type oxygen vacancy states. Annealing as a means of reducing the number of oxygen vacancies improves the rectification ratio, response speed and sensitivity of AlN based photodetectors [15]. Schottky barrier diodes (SBDs) have simple structures to form and yet they can be the basis for complicated device structures such as junction barrier Schottky (JBS) diodes and various types of transistors [16].

In this study, we fabricated AlN/SiC SBDs and investigated the effects of rapid thermal annealing (RTA) on SBD properties.

## 2. Materials and Methods

**Figure 1:**
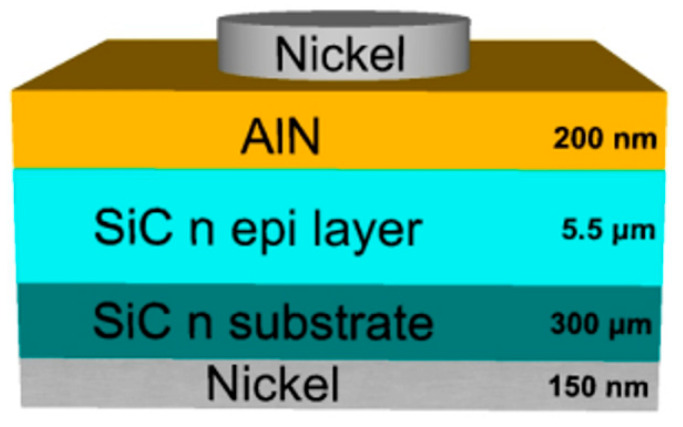
Structure of an aluminum nitride (AlN) Schottky barrier diode.

A schematic of the vertical oxygenated-AlN SBD is shown in Figure 1. The starting substrates were n-type 4H-SiC wafers, onto which AlN thin films were deposited using an RF-sputtering method. AlN films were deposited from an AlN target with 99.9% purity, in an atmosphere of high purity argon gas (99.999%), onto 4H-SiC wafers (N_D_ = 1 × 10^19^ cm^−3^) with an n-type epitaxial 4H-SiC layer (N_D_ = 5 × 10^16^ cm^−3^). After chemical cleaning of the SiC substrate in a 4:1 Sulfuric Peroxide Mix (SPM) cleaning solution of sulfuric acid (H_2_SO_4_) and hydrogen peroxide (H_2_O_2_), the native SiO_2_ layer was stripped using a buffered oxide etch solution. A 150 nm thick Ni-film was then deposited using e-beam evaporation to create a diode cathode on the reverse side of the substrate. After the back-side nickel (Ni)-deposition, the samples were annealed at 1323 K in N_2_ for 90 s by RTA to form nickel silicide (Ni_2_Si) for ohmic contacts. AlN films were then deposited by RF sputtering onto the front of the substrate at room temperature. A sputtering power of 150 W was applied to a 5.08 cm diameter target. Argon gas was injected into the chamber at a flow rate of 5.5 sccm using a mass flow controller. The working pressure was maintained at 10 mTorr during the 120 min deposition time, resulting in a film thickness of approximately 200 nm. The deposited AlN layer was annealed at 673 K, for 5 min in a nitrogen atmosphere. For the top electrode contact, a Ni (150 m) metal layer was deposited. Auger Electron Spectroscopy (AES) was used to analyze the stoichiometry of the AlN films. The SBDs were characterized by current–voltage (I-V) measurements carried out over the temperature range 300–450 K. The J-V characteristics were measured using a semiconductor analyzer (Keithley 4200-SCS) at 300K under ambient air pressure.

## 3. Results and Discussion

**Figure 2:**
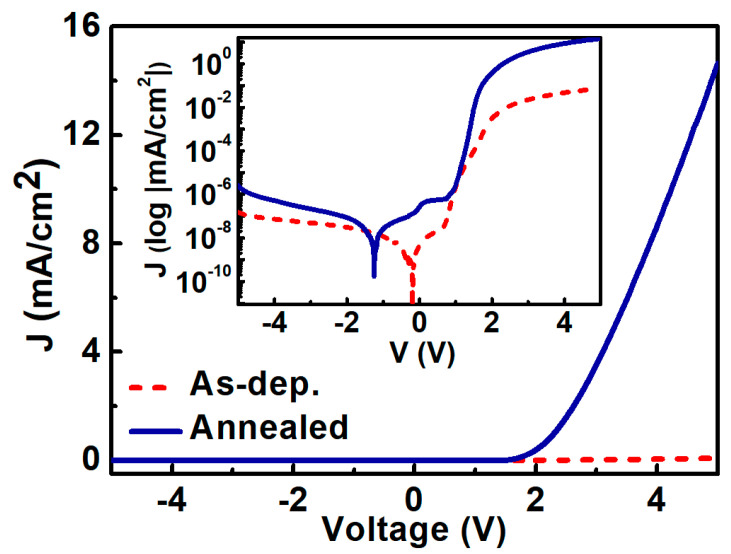
J-V curves of as-deposited (before annealing) and annealed AlN Schottky barrier diodes.

Figure 2 shows the typical current density–voltage (J-V) characteristics of the fabricated oxygenated-AlN SBDs measured at 300 K. The J-V characteristics on a log scale are shown in the inset of Figure 2. The forward current density of the as-deposited device was lower than that of the annealed device (annealed at 673 K for 5 min). In the case of reverse bias, the annealed AlN SBD exhibited a higher leakage current to that of the as-deposited AlN SBD, rising from 1.5 × 10^−7^ to 2.6 × 10^−6^ mA/cm^2^.

**Figure 3:**
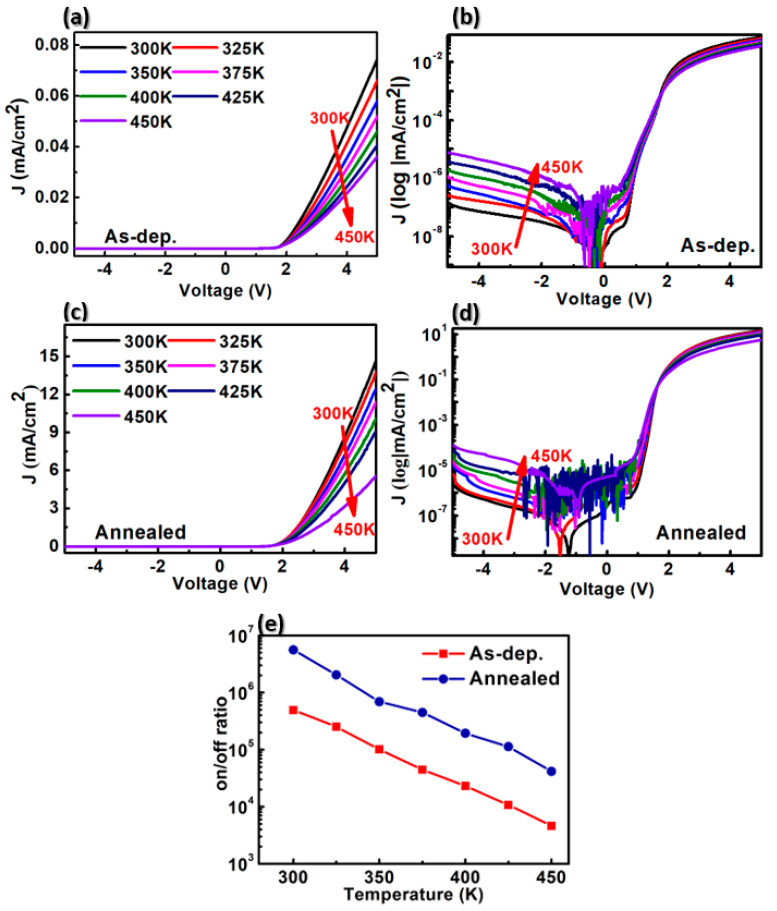
Temperature-dependent J-V curves of as-deposited Schottky barrier diode (SBD) (**a**) linear scale, (**b**) log scale and annealed SBD (**c**) linear scale, (**d**) log scale, (**e**) on/off ratio of fabricated SBDs.

To study how thermal annealing influenced the electrical properties of each AlN SBD, J-V measurements were made for varying temperatures. Figure 3 shows the J-V characteristics of different devices with and without annealing, measured at temperatures between 300 K and 450 K, at steps of 25 K. Both samples exhibit good rectification features, as can be seen from Figure 3b,d. The reverse current density increased with device temperature, while the forward current density decreased. At increasing temperatures, the forward current decreased, owing to the thermionic emission (TE) of the SBDs with series resistance ($R_{s}$). The J-V-T characteristics of the diodes were evaluated according to the TE model [17,18] given by

(1) $$J=J_{s}\left[\exp\left(\frac{q\left(V-IR_{s}\right)}{\eta kT}\right)-1\right]$$

where *q* is the electron charge, $IR_{s}$ is the voltage drop across $R_{s}$, *T* is the measurement temperature, η is the ideality factor, *k* is Boltzmann’s constant, and $J_{s}$ is the saturation current density. $J_{s}$ is the intercept at zero bias of the extrapolated straight-line region of the forward bias current. It is given by

(2) $$J_{s}=A^{*}T^{2}\exp\left(-\frac{q\phi_{B}}{kT}\right)$$

**Figure 4:**
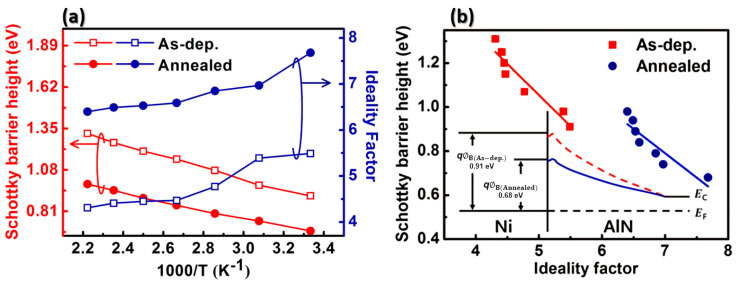
(**a**) Temperature dependence of the Schottky barrier height and ideality factor of each device. (**b**) the Schottky barrier height versus the ideality factor of each device. Inset: the energy band diagram of fabricated devices.

$\phi_{B}$ is the barrier height, and *A*^∗^ is the effective Richardson constant (~57.6 Acm^−2^K^−2^ for AlN) [16]. The temperature effect on I_on_/I_off_ ratio of the fabricated device is shown in Figure 3e, where I_off_, I_on_ is the diode current measured at −5V and +5V bias respectively. The on/off ratios of the as-deposited devices and the annealed devices were calculated to be ~4.9 × 10^5^ and ~5.6 × 10^6^ at room temperature, respectively. The on/off ratio of the annealed device was an order of magnitude (~10 times) higher than the on/off ratio of the as-deposited device over the entire temperature range. Although the off-current increased as the measuring temperature increased, the annealed SBD exhibited a high temperature switching capability with a high on/off ratio of about ~10^5^. After the thermal annealing process at 673 K, the leakage current density of the AlN SBDs increased in reverse bias. However, the Schottky barrier height of the annealed device was lowered in forward bias and the rectifying characteristic was improved. This characteristic is useful to apply to sensor applications. The temperature dependences of the ideality factor and barrier height are shown in Figure 4a. The relationship between barrier height ($\phi_{B})$ and ideality factor (*η*) is determined as the slope of the linear region of the curve depicting the forward bias ln(J)-V characteristics in accordance with

(3) $$\eta =\frac{q}{kT}\left[\frac{dV}{d\left(lnJ\right)}\right]$$

(4) $$\phi_{B}=-\frac{kT}{q}\ln\left(\frac{J_{s}}{A^{*}T^{2}}\right)$$

From Figure 3a,c, the Schottky barrier height *ϕ_B_* and the ideality factor *η* of the manufactured diodes were extracted. The determined values as a function of temperature are shown in Figure 4a,b. The “as-deposited” device had a Schottky barrier height *ϕ_B_* = 0.91 eV and an ideality factor of *η* = 5.49 at room temperature. By contrast, after annealing of the contact at 673 K, a decrease in the barrier height to 0.68 eV was observed, while the ideality factor *η* increased to 7.61. Figure 4b displays a linear relationship between barrier height and the ideality factor. As Figure 4b shows, the lower the ideality factor, the greater the barrier height. The behavior of the barrier height and the ideality factor with increasing temperature is a common feature of Schottky barriers. It is likely to stem from the lack of homogeneity of the metal–semiconductor contact. Inhomogeneity results from a multitude of sources, including varying densities of surface defects, as well as the nature of deposition and surface cleaning processes [19,20]. We assume that the inhomogeneity of the Nickel-AlN contact was reinforced by the RTA process.

**Figure 5:**
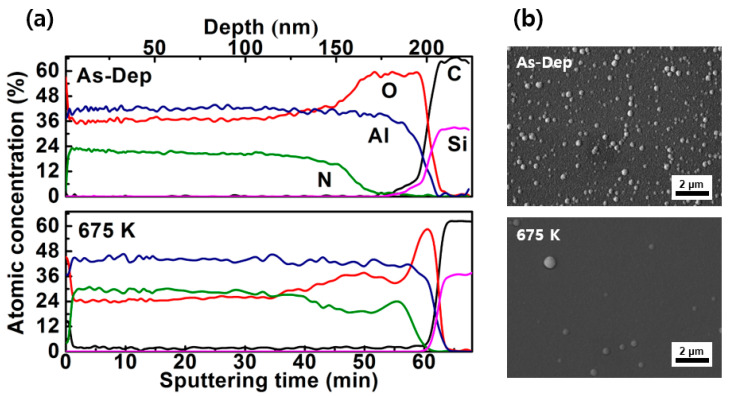
(**a**) Auger Electron Spectroscopy (AES) concentration profile (**b**) SEM images of fabricated AlN /4H-SiC.

The results from AES measurements are shown in Figure 5a. In both devices, the atomic concentration of aluminum was higher than that of nitrogen, from the surface to a depth of ~200 nm into the AlN film layers. During the fabrication of AlN films by the RF sputtering system, the presence of oxidized materials in deep position has been reported [21,22]. Figure 5b shows the SEM micrographs of AlN films on SiC following the As-dep and annealing temperatures. The films exhibited good surface coverage and particles were significantly decreased for the annealed sample [23]. From the aspect of thermo-dynamical data, it can be inferred that Al–O bonds are likely to have formed. This is because ∆G(Al_2_O_3_) = −1480 KJ/mol and ∆G(AlN) = −253 KJ/mol [24]. During the film fabrication, residual oxygen gas in the chamber can react easily with aluminum, forming Al_X_O_Y_ compounds. After the RTA process at 673 K, it was shown that the concentration of aluminum (Al) remained almost the same (~46%), whereas that of oxygen decreased from 36% to 24%. There was an obvious change in the oxygen distribution in AlN SBDs, indicating oxygen loss in the annealed device. It has been reported that, as a result of annealing in a nitrogen atmosphere, the atoms in the film layer may acquire enough kinetic energy to allow them to occupy positions that minimize the number of micro-voids and hence the lattice strain, resulting in improved crystallinity of AlN films [25]. In addition, the unintentionally generated vacancies can be cured by the incorporation of nitrogen atoms [26]. Oxygen is typically observed as a defect in AlN films, which may occupy nitrogen sites and cause aluminum vacancies to equalize the electric charge. It leads to strain misfits and thereby increased phonon scattering, leading to reduced conductivity [27,28,29,30,31]. Consequently, the decreasing atomic concentration of oxygen in the annealed device resulted in a higher electrical conductivity than in the as-deposited device. It was shown that after the RTA process, AlN SBDs had improved electrical conduction properties.

The results shown in Figure 4, together with those in Figure 3, show that rapid thermal annealing treatment of AlN/SiC structures may be important for modifying the behavior of devices by controlling the barrier height and on/off ratio.

## 4. Conclusions

In summary, electrical characteristics and AES were used to investigate the effect of RTA on oxygenated-AlN SBDs. We measured and analyzed the electrical characteristics of an AlN SBD before and after RTA. After the RTA process, the atomic concentration of oxygen in the AlN thin film decreased from ~36% to ~24% and the barrier height decreased from ~0.91 to ~0.68 eV, respectively. The barrier height decreases with improved conductivity, resulting in higher current density values, which in turn results in an improved on/off ratio in the annealed devices. As a result, the RTA process improves the electrical properties of SBDs in AlN/SiC devices.
